# Supplementary material for: Identification of Genes Related to Cold Tolerance and Novel Genetic Markers for Molecular Breeding in Taiwan Tilapia (Oreochromis spp.) via Transcriptome Analysis
Source: Animals (Basel). 2021 Dec 13;11(12):3538. doi: 10.3390/ani11123538 (PMC8697892; doi:10.3390/ani11123538)
Supplement: Supplementary file 1 [file animals-11-03538-s001.zip › animals-1465962-supplementary.pdf]

Supplementary Figure

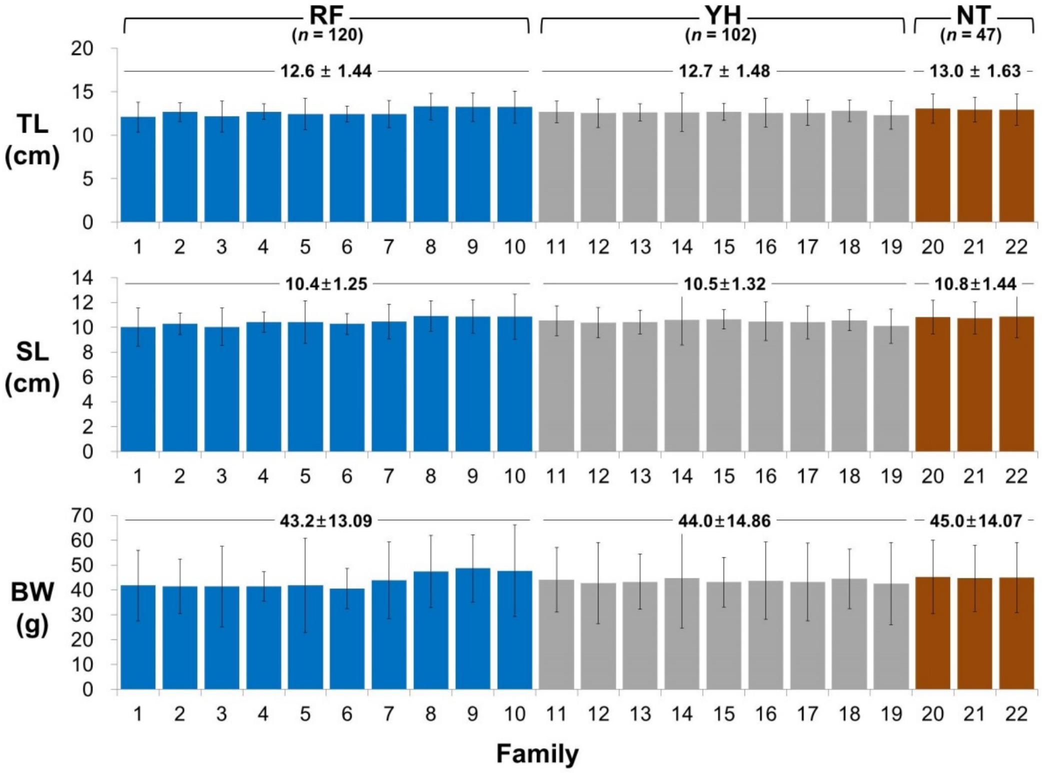

Figure S1. Size statistics of experimental fish family. The TL on the vertical axis refers to the overall length (cm); SL refers to the standard body length (cm); BW refers to the weight (g) and other three measurements are fish size average ± standard deviation. The average TLs of RF (*n* = 120), YH (*n* = 102), and NT (*n* = 47) tested strains were 12.6 ± 1.44 cm, 12.7 ± 1.48 cm, and 13.0 ± 1.63 cm, respectively. The average SLs were 10.4 ± 1.25 cm, 10.5 ± 1.32 cm, and 10.8 ± 1.44 cm, respectively. The average BWs were 43.2 ± 13.09 g, 44.0 ± 14.86 g, and 45.0 ± 14.07 g, respectively.

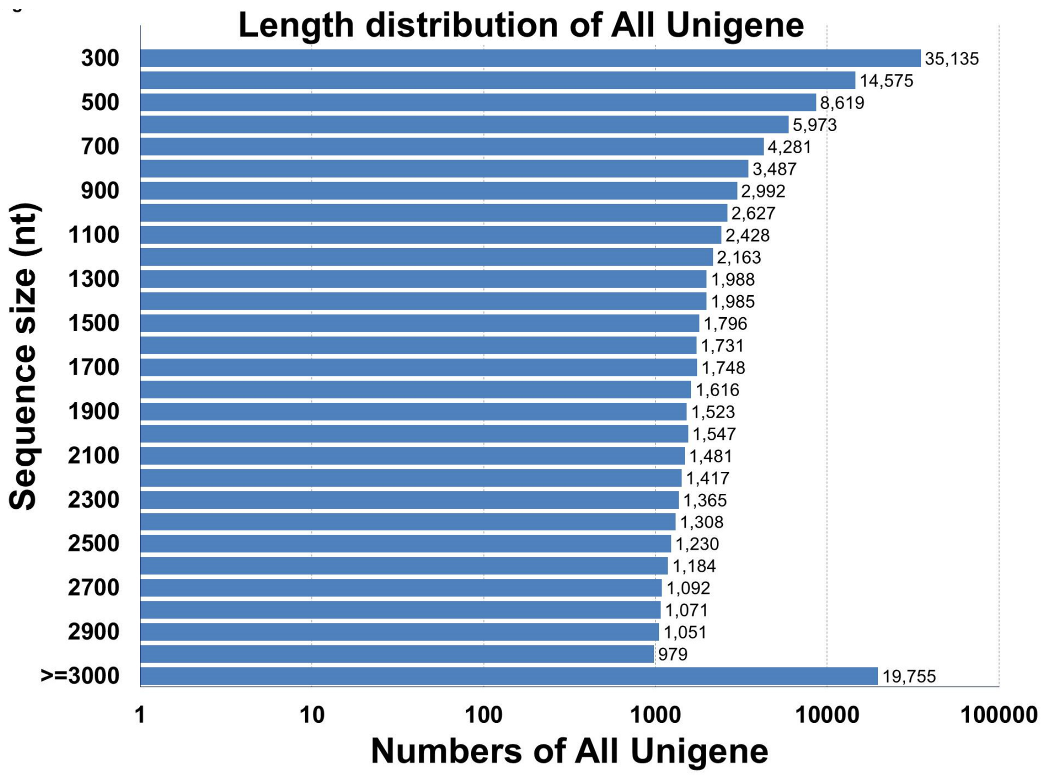

Figure S2. The size distribution of unigenes from four tissues of two tilapia groups. All tissues-unigene. Y-axis represents the length of transcripts. X-axis represents the number of transcripts.

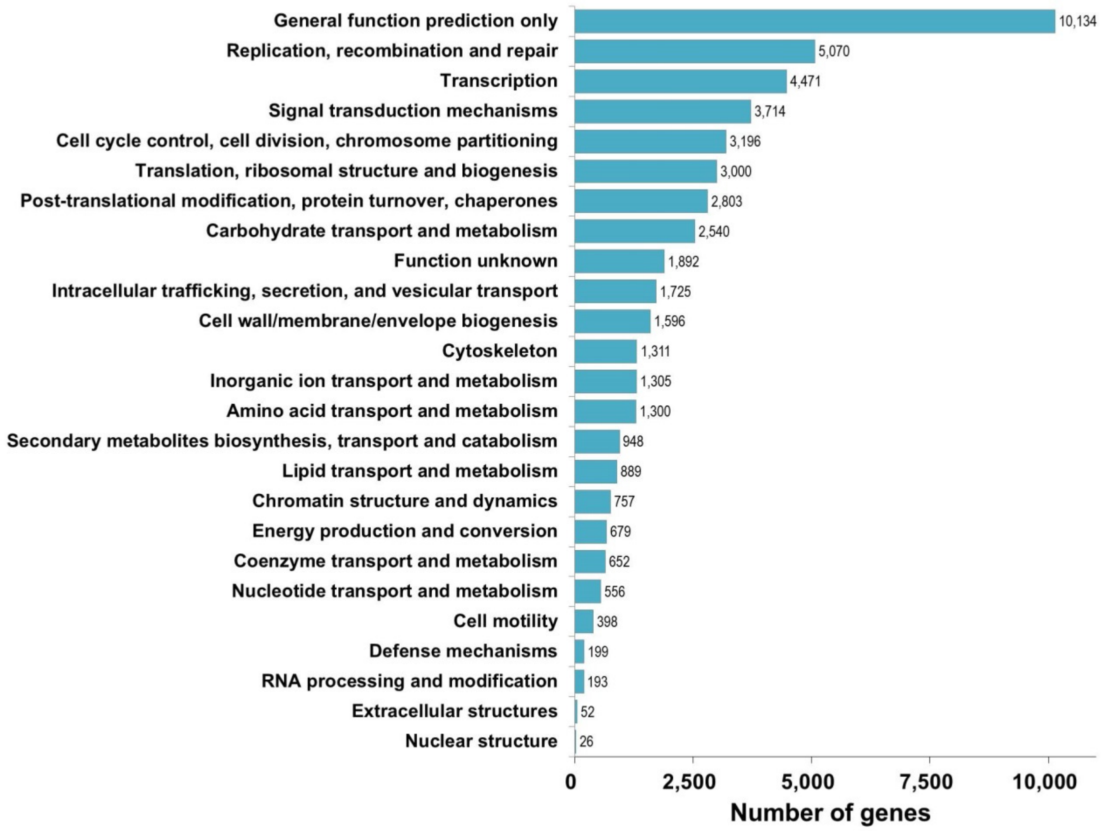

Figure S3. Distribution map of COG function annotation in Taiwan tilapia transcript. X-axis represents the number of unigenes. Y-axis represents the COG functional category.

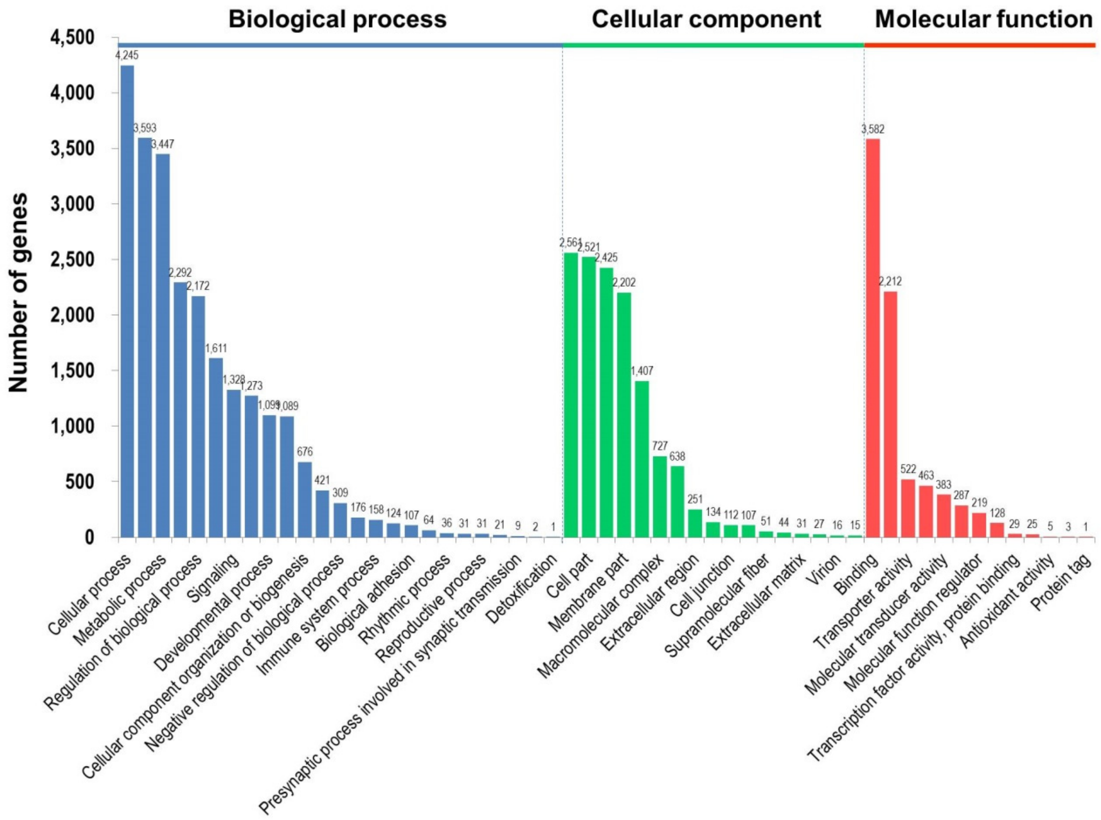

Figure S4. Functional distribution of GO annotation. X-axis represents the number of unigenes. Y-axis represents the Gene Ontology functional category.

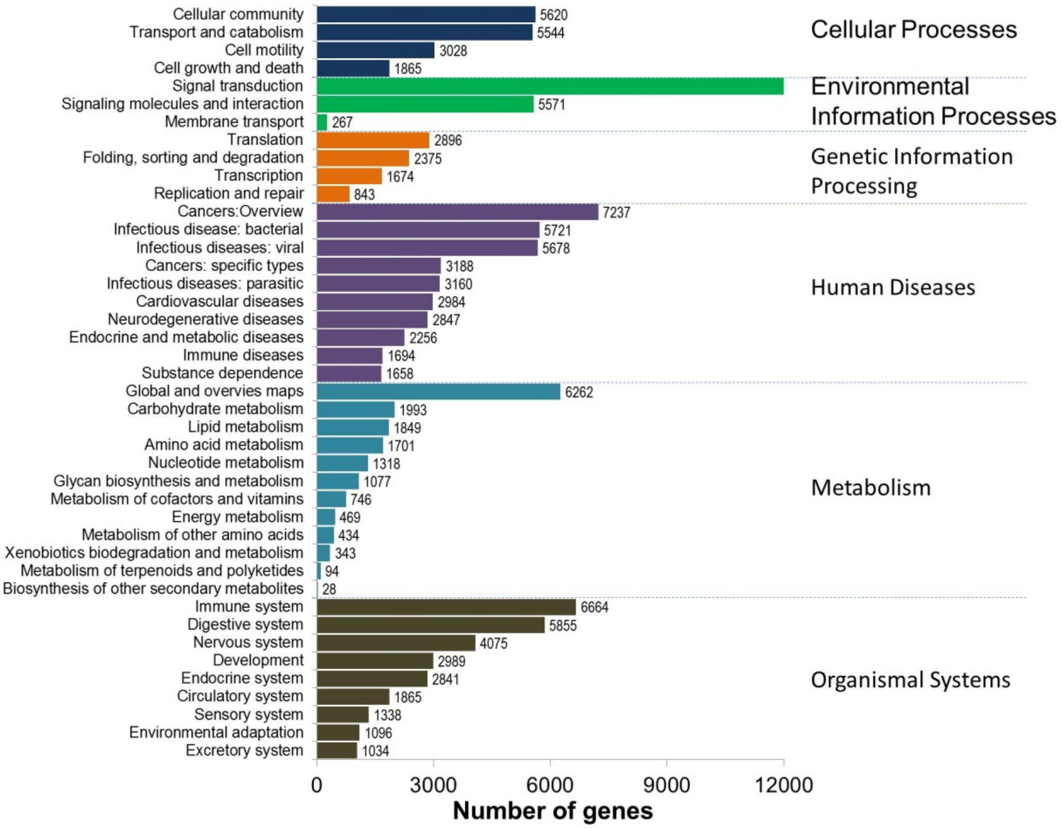

Figure S5. Functional distribution of KEGG annotation. X-axis represents the number of unigenes. Y-axis represents the KEGG functional category.

Table S1

Nucleotide sequences, melting temperature, amplicon size and Ct value of the cold tolerance-related gene primers used for real-time quantitative reverse transcription-PCR assays.

Gene name (GenBank No.)	Forward and Reverse Primer sequences (5'-3')	T_m[1] (℃)	Amplicon size (bp)	Ct value (Mean±SD)
CL10781_10	F: AGCCGTGTTTCTGCATACTA	56	107	16.97±1.73
	R: GTAACTGATGACTGTGTGGTC	55		
CL1487_25	F: AGAGGCACA AAGAAGAATCCA	56	90	19.59±0.97
	R: ACTGCCCACCATTATCAGC	57		
CL5212_1	F: TCTCACATTGCATCAGAAGGAT	56	100	18.90±0.85
	R: TAGGCATCGTTAAAGGCAGG	57		
CL5902_1	F: GTAAAGCGTAAGCAAGTGGG	56	98	18.56±2.33
	R: CCAGACTCCACCCAAACAC	58		
Unigene196	F: CACTACCACCACCTCCTTC	57	102	18.07±0.60
	R: TACCTCGTCCTCCTCCAT	57		
Unigene7071	F: CGGAGGAGGAGGAGTCAG	60	102	19.88±0.95

	R: GAAACACAACAACGCTCGT	56		
18S rRNA (DQ397879)	F: GGCAACCAACGGTAAAACAA	56	84	16.96±0.24
	R: AGCTAGCTGCGTTCTTCATTG	58		
β-actin (EU887951)	F: ACCCACACAGTGCCCATC	61	63	15.06±0.47
	R: CAGGTCCAGACGCAGGAT	60		

[1] The T_m (melting temperature) was calculated for a final primer concentration of 0.2 μM.

Table S2

Quality metrics of transcripts in Taiwan tilapia (*Oreochromis* spp.).

Sample[1]	Number	Length	Mean	N50[2]	N70[3]	N90[4]	GC (%)[5]
Contig							
CT-B	133,054	130,779,739	982	2,246	1,133	326	47.75
CT-G	100,424	83,805,894	834	1,755	850	287	47.86
CT-L	66,700	51,911,379	778	1,518	724	280	48.23
CT-M	51,905	37,118,179	715	1,316	614	264	48.36
CS-B	126,724	128,656,922	1,015	2,313	1,183	340	47.71
CS-G	102,348	85,345,751	833	1,755	842	288	47.19
CS-L	63,638	49,023,003	770	1,499	716	278	47.93
CS-M	54,144	38,682,374	714	1,332	617	262	48.39
Unigene							
CT-B	93,058	111,426,288	1,197	2,586	1,422	413	47.89
CT-G	68,092	70,915,797	1,041	2,039	1,125	372	48.08
CT-L	49,430	45,069,828	911	1,726	880	332	48.42
CT-M	39,415	32,309,325	819	1,493	722	302	48.55
CS-B	88,793	109,168,522	1,229	2,605	1,463	432	47.86
CS-G	69,381	71,761,045	1,034	2,047	1,112	369	47.44
CS-L	47,586	42,421,675	891	1,684	857	323	48.15
CS-M	40,938	33,383,414	815	1,503	715	301	48.53
All	128,147	185,382,926	1,446	3,157	1,920	536	47.46

[1] CT-B, brain tissue of the cold-tolerance group; CT-G, gill tissue of the cold-tolerance group; CT-L, liver tissue of the cold-tolerance group; CT-M, muscle tissue of the cold-tolerance group; CS-B, brain tissue of the cold-sensitive group; CS-G, gill tissue of the cold-sensitive group; CS-L, liver tissue of the cold-sensitive group; CS-M, muscle tissue of the cold-sensitive group.

[2] A weighted median statistic that 50% of the total length is contained in Unigenes great than or equal to this value.

[3] A weighted median statistic that 70% of the total length is contained in Unigenes great than or equal to this value.

[4] A weighted median statistic that 90% of the total length is contained in Unigenes great than or equal to this value.

[5] GC (%): the percentage of G and C bases in all Unigenes.
