# Supplementary material for: Identification of Genes Related to Cold Tolerance and Novel Genetic Markers for Molecular Breeding in Taiwan Tilapia (Oreochromis spp.) via Transcriptome Analysis
Source: Animals (Basel). 2021 Dec 13;11(12):3538. doi: 10.3390/ani11123538 (PMC8697892; doi:10.3390/ani11123538)
Supplement: Supplementary file 1 [file animals-11-03538-s001.zip › Table_S3.pdf]

Table S3

Allele frequency of polymorphic SSR markers in different populations of Taiwan tilapia.

Locus[a]	Allele length (bp)	Allele	Allele frequency			Genotypes		
			RF	YH	NT	RF	YH	NT
UNH916	115	A	0.069	0.000	0.000	115/115, 131/131, 131/149, 131/153, 131/161, 131/177, 131/183, 149/149, 149/153, 149/177, 153/153, 153/161, 153/163, 153/177, 163/177, 173/177	149/149, 149/153, 153/153, 153/159, 153/161, 153/167, 153/177, 159/161, 159/167, 159/177, 161/161, 167/177	149/153, 153/153
	131	B	0.236	0.000	0.000			
	149	C	0.201	0.069	0.266			
	153	D	0.347	0.706	0.734			
	159	E	0.000	0.049	0.000			
	161	F	0.028	0.039	0.000			
	163	G	0.014	0.000	0.000			
	167	H	0.000	0.044	0.000			
	173	I	0.007	0.000	0.000			
	177	J	0.090	0.093	0.000			
	183	K	0.007	0.000	0.000			
UNH999	97	A	0.181	0.015	0.000	97/97, 97/117, 97/121, 97/137, 105/117, 105/137, 111/137, 117/117, 117/133, 117/137, 121/123, 123/137, 127/137, 131/137, 135/135, 135/137, 135/145, 137/137	97/117, 97/137, 105/127, 105/137, 111/137, 113/137, 117/117, 117/127, 117/137, 121/127, 121/137, 123/133, 123/137, 127/127, 127/133, 127/137, 133/137, 135/137, 137/137, 137/151	117/117, 117/137, 137/137
	105	B	0.035	0.015	0.000			
	111	C	0.007	0.005	0.000			
	113	D	0.000	0.005	0.000			
	117	E	0.243	0.093	0.266			
	121	F	0.021	0.034	0.000			
	123	G	0.028	0.020	0.000			
	127	H	0.007	0.275	0.000			
	131	I	0.007	0.000	0.000			
	133	J	0.021	0.034	0.000			
	135	K	0.056	0.005	0.000			
	137	L	0.375	0.495	0.734			
	145	M	0.021	0.000	0.000			
	151	N	0.000	0.005	0.000			
CL1487_25	112	A	0.007	0.000	0.000	112/128, 122/122, 122/126, 122/128, 124/126, 124/128, 124/130, 126/126, 126/128, 126/130, 128/128	122/122, 122/124, 122/128, 124/124, 124/128, 124/132, 128/128, 128/132	128/128
	122	B	0.111	0.270	0.000			
	124	C	0.035	0.387	0.000			
	126	D	0.514	0.000	0.000			
	128	E	0.319	0.324	1.000			
	130	F	0.014	0.000	0.000			
	132	G	0.000	0.020	0.000			
CL1876_16	171	A	0.174	0.000	0.000	171/171, 171/174, 174/174,	174/174	174/174
	174	B	0.826	1.000	1.000			
	162	A	0.014	0.000	0.000	162/164, 162/165, 164/165, 164/167, 164/168, 165/166, 165/168, 165/169, 166/166,	164/164, 164/166, 164/167, 164/168, 164/169, 164/174	
	164	B	0.028	0.294	0.000			
	165	C	0.139	0.005	0.000			
	166	D	0.118	0.064	1.000			

CL5902_1	167	E	0.194	0.485	0.000	[illegible] 166/167, 166/168, 166/169, 167/168, 167/169, 168/168, 168/169, 168/176, 169/169	[illegible] 165/167, 166/166, 166/167, 167/167, 167/168, 168/168	166/166
	168	F	0.417	0.137	0.000			
	169	G	0.083	0.010	0.000			
	174	H	0.000	0.005	0.000			
	176	I	0.007	0.000	0.000			
CL9541_2	168	A	0.319	0.000	0.000	168/168, 168/171, 168/174, 171/174, 174/174	171/171, 171/174, 174/174	171/174, 174/174
	171	B	0.069	0.377	0.223			
	174	C	0.611	0.623	0.777			
CL10781_10	202	A	0.069	0.000	0.000	210/213, 213/217, 213/213, 213/214, 202/202, 209/209, 210/210, 210/214	209/213, 209/214, 213/213, 213/214, 213/217, 214/214, 214/215, 214/217, 215/215	212/212, 213/213, 213/214, 214/214, 215/215
	209	B	0.014	0.015	0.000			
	210	C	0.104	0.000	0.000			
	212	D	0.000	0.000	0.085			
	213	E	0.757	0.485	0.436			
	214	F	0.035	0.412	0.457			
	215	G	0.000	0.015	0.021			
	217	H	0.021	0.074	0.000			
	179	I	0.014	0.240	0.521			
CL279_7	223	A	0.931	1.000	1.000	223/223, 229/229	223/223	223/223
	229	B	0.069	0.000	0.000			
CL2262_1	151	A	0.194	0.034	0.000	151/151, 151/154, 151/156, 151/158, 151/159, 151/160, 152/153, 153/156, 153/158, 153/159, 154/158, 154/159, 156/158, 156/159, 158/158, 158/159, 158/160, 158/161, 158/162, 159/159, 159/160, 160/162, 161/161	151/156, 151/158, 151/160, 156/156, 156/158, 156/160, 156/161, 158/158, 158/160, 158/161, 159/161, 160/160, 160/161, 161/161	158/158, 158/159, 158/162, 159/160, 159/162
	152	B	0.007	0.000	0.000			
	153	C	0.042	0.000	0.000			
	154	D	0.035	0.000	0.000			
	156	E	0.035	0.363	0.000			
	158	F	0.236	0.172	0.372			
	159	G	0.319	0.005	0.309			
	160	H	0.076	0.319	0.011			
	161	I	0.021	0.108	0.000			
	162	J	0.035	0.000	0.309			
Unigene7071	126	A	0.069	0.000	0.000	126/126, 128/128, 128/144, 128/148, 128/152, 128/154, 128/156, 128/158, 144/152, 144/156, 148/152, 148/156, 152/152, 152/154, 152/156, 154/156, 156/156, 156/158, 156/164	128/144, 128/156, 142/156, 144/154, 144/156, 152/156, 154/156, 156/156	128/128, 128/154, 154/154
	128	B	0.229	0.054	0.691			
	142	C	0.000	0.005	0.000			
	144	D	0.042	0.025	0.000			
	148	E	0.063	0.000	0.000			
	152	F	0.111	0.010	0.000			
	154	G	0.035	0.015	0.309			
	156	H	0.424	0.892	0.000			
	158	I	0.021	0.000	0.000			
	164	J	0.007	0.000	0.000			
	187	A	0.354	0.157	0.000			
	193	B	0.014	0.000	0.340			
	197	C	0.083	0.368	0.000			

Unigene196	199	D	0.028	0.000	0.000	187/187, 187/193, 187/197, 187/199, 187/203, 187/205, 187/211, 187/215, 187/229, 193/199, 197/203, 197/205, 197/207, 197/213, 197/215, 199/203, 203/203, 203/205, 203/207, 203/211, 203/215, 203/219, 203/229, 205/209	187/187, 187/197, 187/207, 187/213, 187/217, 197/197, 197/207, 197/213, 197/215, 197/217, 197/219, 207/207, 207/213, 207/217	193/193, 193/211, 211/211
	203	E	0.292	0.000	0.000			
	205	F	0.076	0.000	0.000			
	207	G	0.014	0.358	0.000			
	209	H	0.007	0.000	0.000			
	211	I	0.035	0.000	0.660			
	213	J	0.007	0.059	0.000			
	215	K	0.056	0.005	0.000			
	217	L	0.000	0.049	0.000			
	219	M	0.014	0.005	0.000			
	229	N	0.021	0.000	0.000			
CL9318_1	158	A	0.069	0.000	0.000	158/158, 176/185, 176/191, 176/194, 176/203, 185/185, 185/188, 185/191, 185/194, 185/200, 185/203, 191/191, 191/203, 194/203, 200/203, 203/203	182/191, 182/215, 188/191, 188/212, 191/191, 191/203, 191/212	191/191
	176	B	0.069	0.000	0.000			
	182	C	0.000	0.064	0.000			
	185	D	0.403	0.000	0.000			
	188	E	0.007	0.039	0.000			
	191	F	0.194	0.843	1.000			
	194	G	0.056	0.000	0.000			
	200	H	0.021	0.000	0.000			
	203	I	0.181	0.005	0.000			
	212	J	0.000	0.044	0.000			
	215	K	0.000	0.005	0.000			
CL241_10	167	A	0.076	0.000	0.000	167/167, 167/173, 173/173, 173/176, 173/179	173/173, 173/179, 179/179	173/173, 173/179, 179/179
	173	B	0.771	0.760	0.479			
	176	C	0.139	0.000	0.000			
	179	D	0.014	0.240	0.521			
